# Enhancement of the cytotoxicity of radiosensitizers by modest hyperthermia: the electron-affinity relationship.

**DOI:** 10.1038/bjc.1982.301

**Published:** 1982-12

**Authors:** S. Rajaratnam, G. E. Adams, I. J. Stratford, C. Clarke

## Abstract

The cytotoxicity of 3 electron-affinic radiosensitizers has been studied in Chinese hamster V-79 cells as a function of pH and modest hyperthermia. When equitoxic concentrations were used and temperature was increased from 34 to 41 degrees C metronidazole, the compound with the lowest electron affinity showed the greatest enhancement of hypoxic-cell toxicity, and nitrofurantoin, the compound with the highest electron affinity, the least. The results can be explained if the mechanisms of toxicity involves a redox reaction, since it would be expected that the least toxic compound (lowest electron affinity) would have the largest activation energy and hence the greatest temperature effect. This appears to hold for these 3 compounds. Experiments also showed that nitrofurantoin which exhibits no increase in toxicity when the temperature was increased from 37 to 41 degrees C at pH 7.4, showed an increase in toxicity for the same temperature change at the pH of 7.0 and 6.6. Under aerobic conditions only metronidazole showed significant toxicity at 41 degrees C, where the differential between aerobic and hypoxic cell toxicity was minimal, both at pH 7.4, and at the low pH values of 7.0 and 6.6. In the clinical setting there is evidence that tumour cells are at a lower pH than their surrounding normal tissues. Hypoxic-cell cytotoxicity is enhanced at low pH, and even further enhanced at low pH in combination with a temperature of 41 degrees C. However, this finding correlates conversely with electron affinity. Thus, the radiosensitizer (and trichomonicide) metronidazole is most influenced by low pH and high temperature with the nitroimidazole, misonidazole, demonstrating a smaller enhancement due to higher temperatures.


*Br. J. Cancer* (1982) **46**, 912

### ENHANCEMENT OF THE CYTOTOXICITY OF RADIOSENSITIZERS BY MODEST HYPERTHERMIA: THE ELECTRON-AFFINITY RELATIONSHIP

S. RAJARATNAM*, G. E. ADAMS, I. J. STRATFORD AND C. CLARKE

*From the Institute of Cancer Research, Sutton, Surrey, England*

Received 19 January 1982 Accepted 10 August 1982

**Summary.—The cytotoxicity of 3 electron-affinic radiosensitizers has been studied in Chinese hamster V-79 cells as a function of pH and modest hyperthermia. When equitoxic concentrations were used and temperature was increased from 34 to 41°C metronidazole, the compound with the lowest electron affinity showed the greatest enhancement of hypoxic-cell toxicity, and nitrofurantoin, the compound with the highest electron affinity, the least. The results can be explained if the mechanisms of toxicity involves a redox reaction, since it would be expected that the least toxic compound (lowest electron affinity) would have the largest activation energy and hence the greatest temperature effect. This appears to hold for these 3 compounds.**

**Experiments also showed that nitrofurantoin which exhibits no increase in toxicity when the temperature was increased from 37 to 41°C at pH 7·4, showed an increase in toxicity for the same temperature change at the pH of 7·0 and 6·6.**

**Under aerobic conditions only metronidazole showed significant toxicity at 41°C, where the differential between aerobic and hypoxic cell toxicity was minimal, both at pH 7·4, and at the low pH values of 7·0 and 6·6.**

**In the clinical setting there is evidence that tumour cells are at a lower pH than their surrounding normal tissues. Hypoxic-cell cytotoxicity is enhanced at low pH, and even further enhanced at low pH in combination with a temperature of 41°C. However, this finding correlates conversely with electron affinity. Thus, the radiosensitizer (and trichomonicide) metronidazole is most influenced by low pH and high temperature with the nitroimidazole, misonidazole, demonstrating a smaller enhancement due to higher temperatures.**

IT HAS BEEN DETERMINED that electron-affinic radiosensitizers are more toxic to hypoxic than aerated cells (Hall & Roizin-Towle, 1975; Mohindra & Rauth, 1976). The toxicity of one such drug, misonidazole (MISO), is a function of drug concentration and contact time (Hall & Roizin-Towle, 1975; Moore *et al.*, 1976; Stratford & Adams, 1977), pH (Stratford, 1979), serum concentration and serum source (Stratford & Gray, 1978; Hall *et al.*, 1977; Whitmore *et al.*, unpublished data) and temperature (Stratford & Adams, 1977; Stratford *et al.*, 1978; Hall *et al.*, 1977).

There is now considerable evidence that both human and rodent tumours are at a lower pH than their surrounding normal tissues (Eden *et al.*, 1955; Ashby, 1966; Kahler & Robertson, 1945). It is more than likely, then, that the hypoxic regions in tumours are also at a low pH. It was therefore of interest to compare the effects of low pH, and low pH with temperature, on the hypoxic-cell toxicity of radiosensitizers.

The hypoxic-cell toxicity of nitro compounds is related to their electron affinity, the more electron-affinic drugs being more cytotoxic (Adams *et al.*, 1980). This

* Present address: Radiological Research Laboratory, Columbia University College of Physicians and Surgeons, 630 West 168th St, New York, NY 10032.

relationship suggests that the mechanism(s) of action involves electron-transfer redox processes. The more toxic compounds with their higher electron affinities should have lower activation energies and hence lower thermal enhancement ratios. This premise was tested.

Three compounds were chosen because of their varying electron affinities as measured by their one-electron reduction potential. They were metronidazole (METRO) $E\frac{1}{7} = -486$ mV, MISO $E\frac{1}{7} = -389$ mV, and nitrofurantoin (NFT) $E\frac{1}{7} = -264$ mV. Since their hypoxic-cell toxicity is markedly different, the effects of pH and temperature were tested at equitoxic concentrations. The concentrations were chosen such that cell survival was reduced to $10^{-2}$ during a 6h period under hypoxia at 41°C. Thus METRO was used at a concentration of 30 mM, MISO at 2 mM and NFT at 100 μM.

Results obtained in this study showed that enhancement of cytotoxicity by temperature was electron-affinity dependent. For a temperature increase from 34 to 41°C METRO, the compound with the lowest electron affinity showed the greatest thermal enhancement and NFT, the compound with the lowest electron affinity, the least.

### MATERIALS AND METHODS

The culture of V79-379A cells and the procedures for carrying out hypoxic-cell toxicity experiments have been described elsewhere (Stratford & Adams, 1977). Briefly, V79-379A cells obtained from an ansynchronous exponential cell suspension were centrifuged and resuspended in growth medium (Eagle's Minimum Essential Medium containing 7·5% foetal calf serum) containing drug at the required concentration. The cells were maintained in conical flasks modified with a side arm through which samples could be withdrawn. For hypoxic experiments, the conical flasks were fitted with Dreschel heads. Hypoxia was maintained by passing 95% $N_2$/5% $CO_2$ (BOC < 10 ppm oxygen) over the surface of the stirred cell suspension. At intervals aliquots of cells were withdrawn *via* the side arm and the number of surviving cells determined. Results shown represent those obtained from at least 3 (and up to 5) replicate experiments.

Modification to pH of the growth medium was obtained by varying the bicarbonate concentration. Values were checked using a PHM64 pH meter (V. A. Howe Ltd) and replicate measurements showed an accuracy of ±0·02. MISO and METRO were donated by Roche Products Ltd and May and Baker Ltd respectively. NFT was purchased from Sigma.

### RESULTS

### *Aerobic toxicity of radiosensitizers: effects of low pH at* 37 *and* 41°C

Control cells showed a decrease in growth rate, but no loss in cell survival when the pH of the extracellular medium was decreased from 7·4 to 6·4 for a period of 60 h at 37°C. The presence of each of the drugs at the concentration chosen caused no additional effects.

The effect of low pH on cell viability at 41°C is shown in Fig. 1 (control) where only at pH 6·4 is there a substantial effect on cell survival, with 70% of cells being capable of colony formation after 9 h but only 0·001% after 24 h. Cells incubated with 2mM MISO or 100μM NFT at 41°C and at the low pH values showed no difference from that obtained in control cells in the 24 h (not shown).

However, there was a marked difference in the results obtained with 30mM METRO (Fig. 1). At 41°C and pH 6·4 only a 4h treatment was sufficient to reduce survival to 0·001%. This contrasts with the 24h treatment time for control, 2mM MISO or 100μM NFT-treated cells, to achieve the same survival level.

### *The hypoxic-cell toxicity of radiosensitizers: potentiation by hyperthermia and the electron-affinity relationship*

Experiments were carried out to determine survival after various times of incubating cells with 30mM METRO, 2mM MISO and 100μM NFT at 34, 37 and 41°C under hypoxic conditions. Results are presented in Fig. 2. It can be seen that the toxicity of both MISO and METRO increased as temperature increased. In

FIG. 1.—The effect of low pH on survival of aerobic cells at 41 °C (left panel) and the effect of 30mM METRO and low pH on aerobic cells at 37 and 41 °C (right panel).

FIG. 2.—The electron-affinity relationship for the thermal enhancement of cytotoxicity. When approximately equitoxic concentrations are used, the effect of higher temperatures is greater for METRO ($E^1_7 = -486$mV) than MISO ($E^1_7 = -389$mV) or NFT ($E^1_7 = -264$mV).

contrast an increase in temperature from 34 to 41°C had no effect on the cytotoxic effect of NFT.

Survival was reduced to $10^{-2}$ in 6 h in the case of both METRO and MISO at 41°C. However, to achieve the same level of survival at 37°C, 12 h was required in the case of METRO and 8 h with MISO. It can be seen that the relative enhancement of hypoxic-cell toxicity by temperature was greater for METRO than for MISO. The enhancement of cytotoxicity by temperature is electron-affinity-dependent. At equitoxic concentrations, METRO had the greatest temperature and NFT the least.

*The enhancement of cytotoxicity by low pH at* 37 *and* 41°*C*

The effects of lowering extracellular pH from pH 7·4 to 7·0 and 6·6 on the hypoxic toxicity of 30mM METRO, 2mM MISO and 100μM NFT (at 37°C) can be seen in Fig. 3; where in every instance lowering the extracellular pH enhanced hypoxic cell toxicity. These pH values were chosen because a pH of 6·4 in the presence of the drug was found to be extremely toxic in every instance.

Fig. 3 (open symbols) shows the effect of the same three extracellular pH values on drug cytotoxicity at 41°C. In conjunction with the greater cytotoxicity due to the increase in temperature, further enhancements in toxicity are apparent when pH is reduced from pH 7·4 to 7·0 and to 6·6.

Hypoxic control cells showed no loss of plating efficiency either at pH 7·4 or at the lower pH values of 7·0 or 6·6 over the duration of these experiments, both at 37° and at 41°C.

At the lowest pH values of 7·0 and 6·6 there was an enhancement of drug cytotoxicity by temperature in every instance, even in the case of NFT where there was no enhancement of toxicity by temperature at pH 7·4.

DISCUSSION

Effects of pH are relevant to studies of the cytotoxic effects of these compounds in the context of applications of these drus *in vivo*. It is known that, in the necrotic regions of tumours near which hypoxic cells are likely to be present, there is substantial drop in the pH of extracellular fluid. This is due to the production of large quantities of lactic acid following anaerobic glycolysis in cells with a low oxygen

FIG. 3.—The effect of reduced pH on the hypoxic-cell cytotoxicity of 30mM METRO (A), 2mM MISO (B) and 100 μM NFT (C) at 37 and 41°C.

supply (von Ardenne, 1972). It has been shown that, *in vitro*, Chinese hamster cells can be maintained in hypoxic suspension culture for long periods without any loss of colony-forming ability even when the pH of the medium is reduced to 6·6 (Stratford, 1979). It has been demonstrated that at 37°C a reduction in the pH of the medium from 7·4 to 6·6 causes a very large increase in the cytotoxic effect of MISO on hypoxic cells of this Chinese hamster cell line (Stratford, 1979). These results were confirmed in this study and extended to determine the effect of pH and hyperthermia on 3 radiosensitizers. Clearly, the pH effect is marked at both 37 and 41°C even for NFT, where there is *no* hyperthermic potentiation over this small temperature range.

The electron-affinity correlation for cytotoxicity suggests that the mechanisms of the metabolic reduction of the drugs involve electron-transfer processes. The concentration-time dependence of hypoxic cytotoxicity implies that the critical reactions involved will have appreciable activation energies. This is in contrast to the extremely fast free radical reactions observed in radiosensitization. The large effect of small temperature changes on cellular inactivation rate is therefore to be expected.

It would follow, therefore, that as the electron affinity increases the rate of metabolic reduction also increases. The activation energy would presumably fall and, if Arrhenius kinetics were to hold, the temperature coefficient of the cytotoxic response would also fall. This premise was tested and found to be true. Three drugs of widely differing electron affinities were used, and the increase in cytotoxicity when temperature was increased from 34 to 41°C was determined. At equitoxic concentration, METRO, the compound with the lowest electron affinity, showed the highest thermal enhancement and NFT, the compound with the lowest electron affinity, the least.

The possibility of increasing the effectiveness of several chemotherapeutic agents by the use of heat has been suggested, and reported in several *in vitro* systems (Hahn, 1978; Roizin-Towle *et al.*, 1982). The results presented in this paper show that the hypoxic-cell toxicity of electron-affinic radiosensitizers can be enhanced by both temperature and low pH, and the degree of thermal enhancement obtained correlated with the electron affinity of the compound. Further, it has been shown that for METRO a small increase in temperature produced a large enhancement of aerobic toxicity. In particular, the large differential between aerobic and hypoxic toxicity usually observed with radiosensitizers was minimal for this drug at 41°C both at the normal pH (7·4) and at the lower pH values tested. This finding warrants further study since it may indicate a deleterious effect of this widely used therapeutic agent when used in conjunction with whole-body hyperthermia in the clinical setting.

### REFERENCES

Adams, G. E., Stratford, I. J., Wallace, R. G., Wardman, P. & Watts, M. E. (1980) Toxicity of nitro compounds toward hypoxic mammalian cells *in vitro*: Dependence on reduction potential. *J. Natl Cancer Inst.* **64**, 555.

von Ardenne, M. (1972) Selective multiphase cancer therapy: Conceptual aspects and experimental basis. *Adv. Pharmacol.*, **10**, 339.

Ashby, B. S. (1966) pH studies in human malignant tumours. *Lancet*, ii, 312.

Eden, H., Haines, B. & Kahler, H. (1955) The pH of rat tumors measured *in vivo*. *J. Natl Cancer. Inst.*, **16**, 541.

Hahn, G. M. (1978) Interaction of drugs and hyperthermia *in vitro* and *in vivo*. In *Cancer Therapy by Hyperthermia and Radiation* (Eds. Streffer *et al.*). Baltimore: Urban and Schwarzenberg.

Hall, E. J., Astor, M., Geard, C. & Biaglow, J. (1977) Cytotoxicity of RO-07-0582. Enhancement by hyperthermia and protection by cysteamine. *Br. J. Cancer*, **35**, 809.

Hall, E. J. & Roizin-Towle, L. (1975) Hypoxic sensitizers: Radiobiological studies at the cellular level. *Radiology*, **117**, 453.

Kahler, H. & Robertson, W. (1945) Hydrogen-ion concentration of normal liver and hepatic tumors. *J. Natl Cancer Inst.*, **3**, 495.

Mohindra, J. & Rauth, A. M. (1976) Increased cell killing by metronidazole and nitrofurazone of hypoxic compared to aerobic mammalian cells. *Cancer Res.*, **36**, 930.

Moore, B. A., Palcic, B. & Skarsgard, L. D. (1976). Radiosensitization and toxic effects of the 2-nitroimidazole RO-07-0582 in hypoxic mammalian cells. *Radiat. Res.*, **67**, 459.

ROIZIN-TOWLE, L., HALL, E. J. & CAPUANO, L. (1982) The interaction of hyperthermia and cytotoxic agents. *J. Natl Cancer Inst.*, (in press).

STRATFORD, I. J. (1979) Cellular radiosensitization: Principles and method of study. In *Radiosensitizers of Hypoxic Cells* (Eds. Breccia *et al.*). Amsterdam: Elsevier North-Holland Biomedical Press. p. 109.

STRATFORD, I. J. & GRAY, P. (1978) Some factors affecting the specific toxicity of misonidazole towards hypoxic mammalian cells. *Br. J. Cancer*, **37**, (Suppl. III) 129.

STRATFORD, I. J., WATTS, M. E. & ADAMS, G. E. (1978) The effect of hyperthermia on the differential cytotoxicity of some electron affinic radiosensitizers on mammalian cells *in vitro*. In *Cancer Therapy by Radiation and Hyperthermia*, (Eds Streffer *et al.*). Baltimore: Urban and Schwarzenberg. p. 267.

STRATFORD, I. J. & ADAMS, G. E. (1977) Effect of hyperthermia on differential cytotoxicity of a hypoxic cell radiosensitizer RO-07-0582, on mammalian cells *in vitro*. *Br. J. Cancer*, **35**, 307.


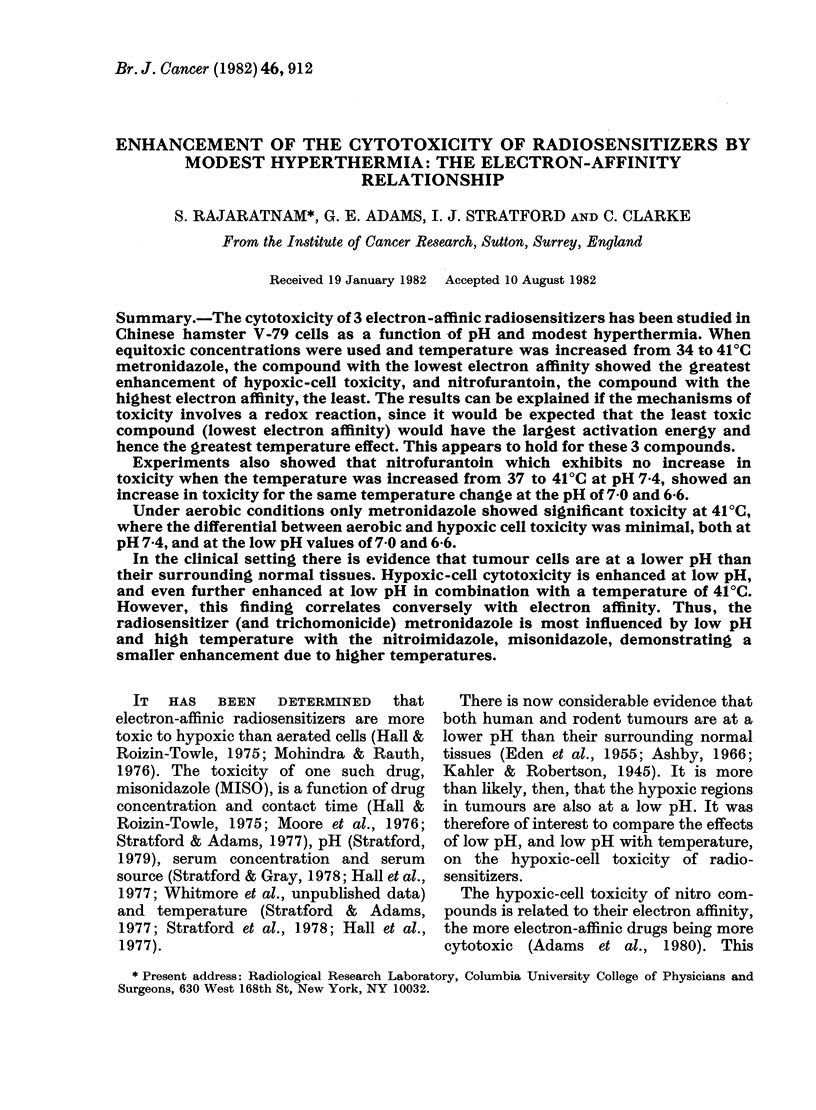

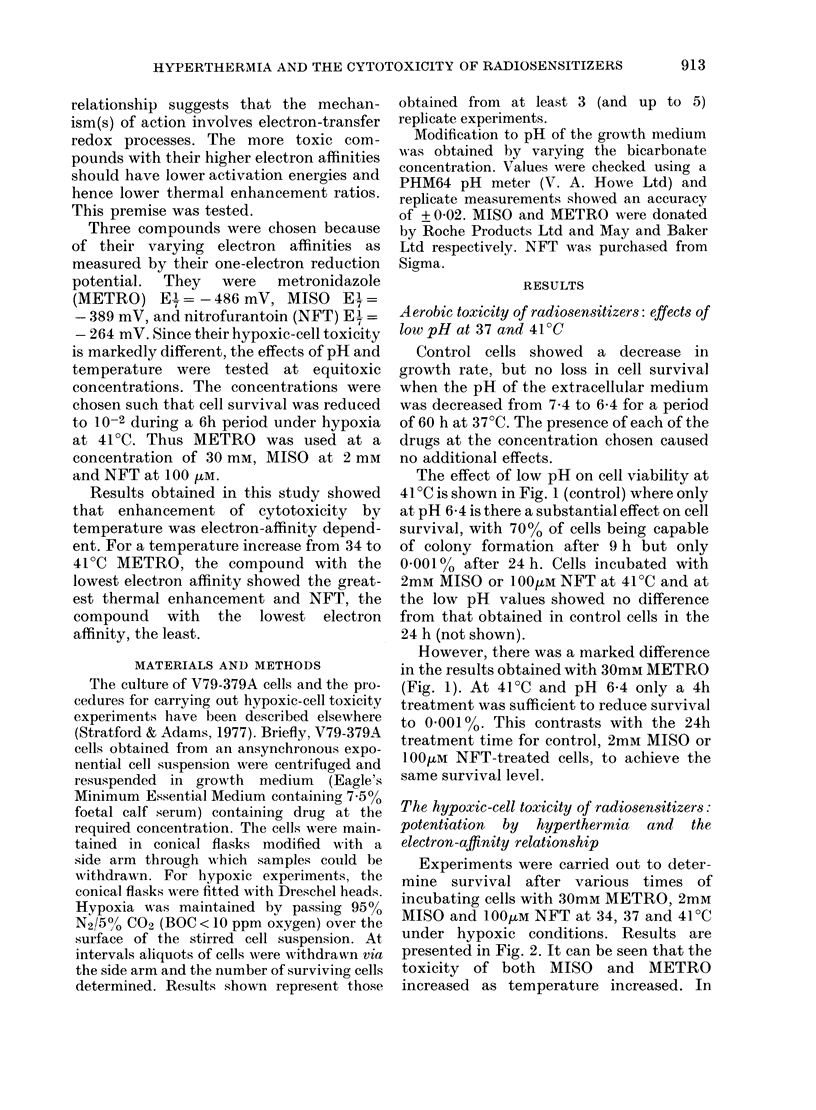

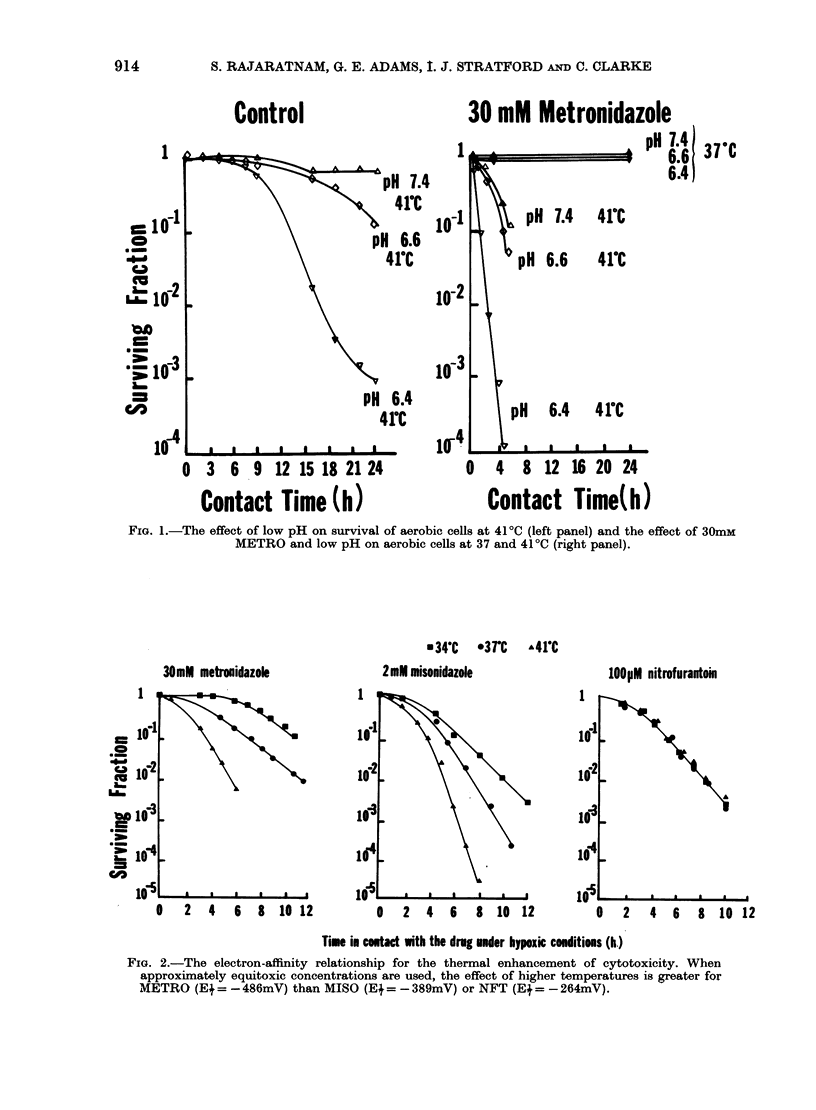

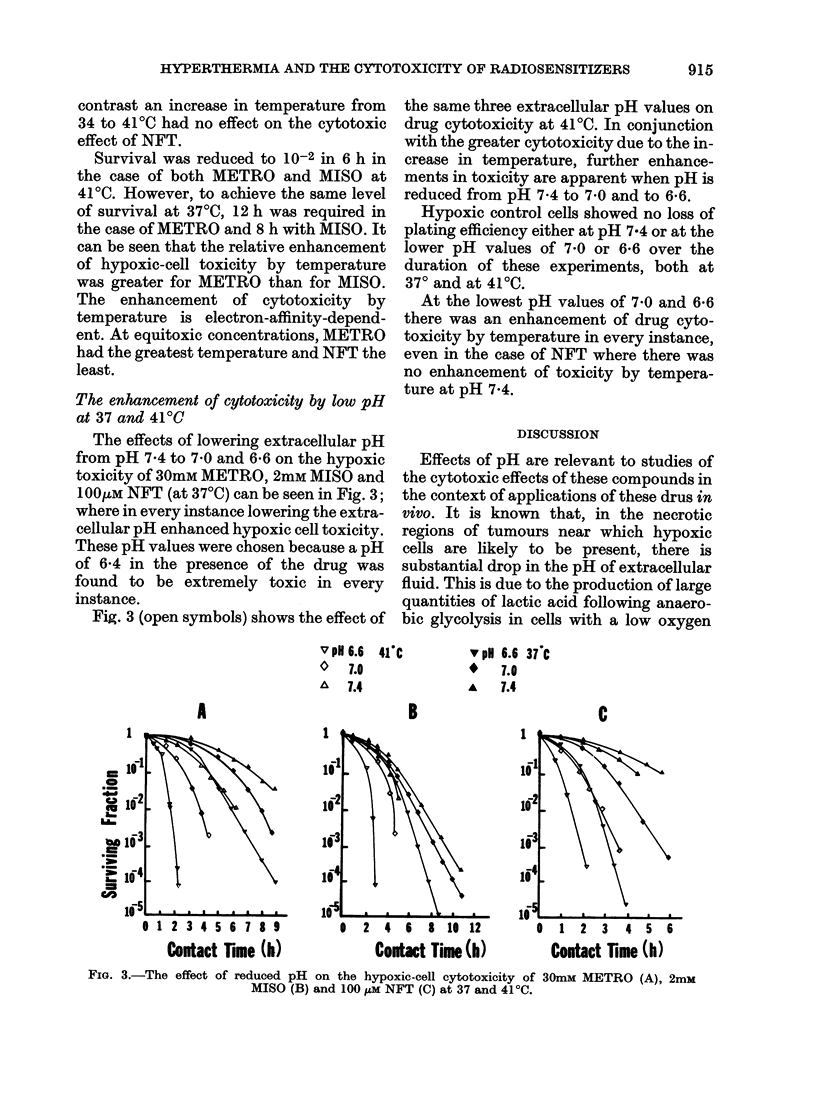

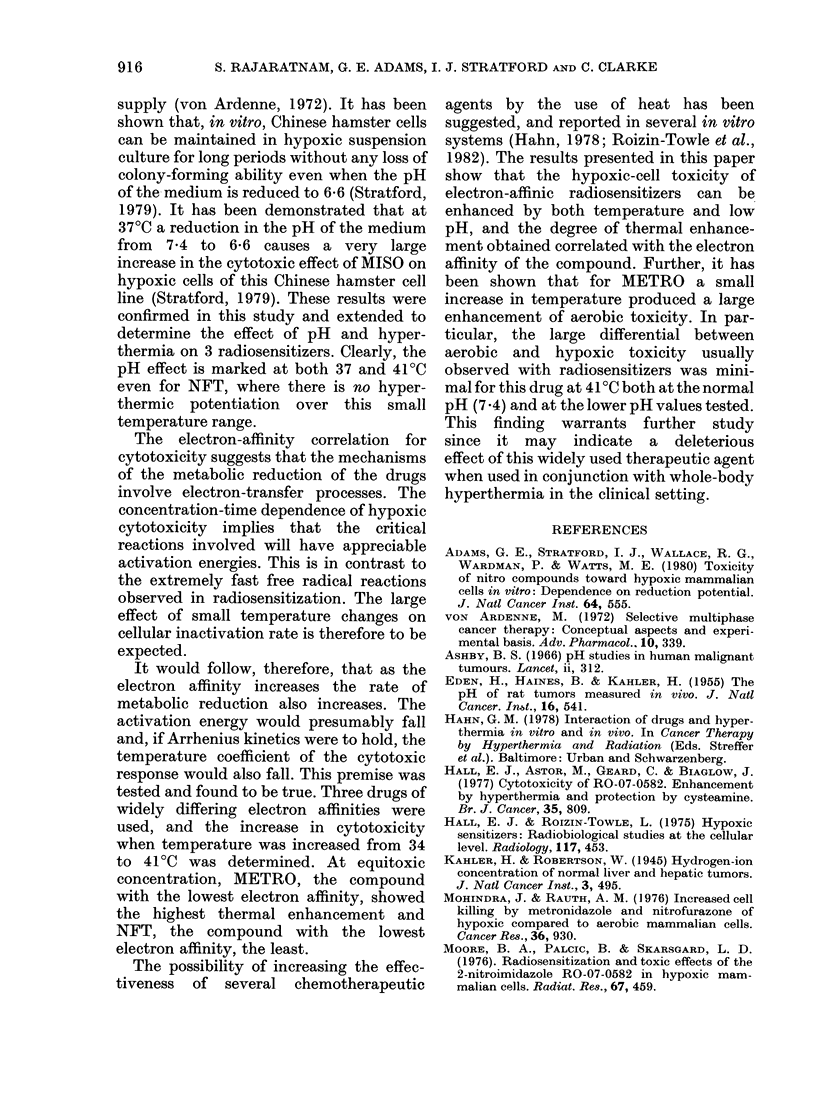

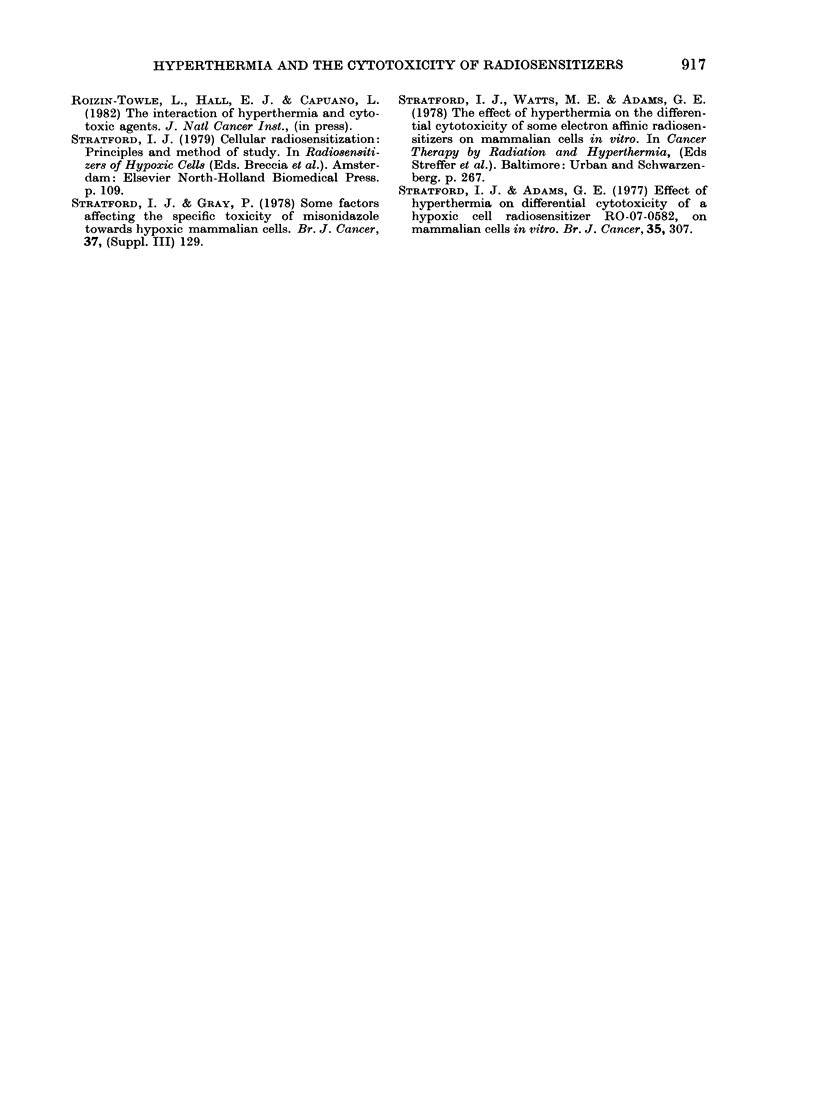


## References

[OCR_00462] Adams G. E., Stratford I. J., Wallace R. G., Wardman P., Watts M. E. (1980). Toxicity of nitro compounds toward hypoxic mammalian cells in vitro: dependence on reduction potential.. J Natl Cancer Inst.

[OCR_00474] Ashby B. S. (1966). pH studies in human malignant tumours.. Lancet.

[OCR_00478] EDEN M., HAINES B., KAHLER H. (1955). The pH of rat tumors measured in vivo.. J Natl Cancer Inst.

[OCR_00489] Hall E. J., Astor M., Geard C., Biaglow J. (1977). Cytotoxicity of Ro-07-0582; enhancement by hyperthermia and protection by cysteamine.. Br J Cancer.

[OCR_00495] Hall E. J., Roizin-Towle L. (1975). Hypoxic sensitizers: radiobiological studies at the cellular level.. Radiology.

[OCR_00513] Moore B. A., Palcic B., Skarsgard L. D. (1976). Radiosensitizing and toxic effects on the 2-nitroimidazole Ro-07-0582 in hypoxic mammation cells.. Radiat Res.

[OCR_00546] Stratford I. J., Adams G. E. (1977). Effect of hyperthermia on differential cytotoxicity of a hypoxic cell radiosensitizer, Ro-07-0582, on mammalian cells in vitro.. Br J Cancer.

[OCR_00531] Stratford I. J., Gray P. (1978). Some factors affecting the specific toxicity of misonidazole towards hypoxic mammalian cells.. Br J Cancer Suppl.

[OCR_00469] von Ardenne M. (1972). Selective multiphase cancer therapy: conceptual aspects and experimental basis.. Adv Pharmacol Chemother.

